# Transcranial Doppler to detect right‐to‐left shunt in cryptogenic acute ischemic stroke

**DOI:** 10.1002/brb3.1091

**Published:** 2018-12-01

**Authors:** Paola Palazzo, Pierre Ingrand, Pierre Agius, Rafik Belhadj Chaidi, Jean‐Philippe Neau

**Affiliations:** ^1^ Department of Neurology Poitiers University Hospital Poitiers Cedex France; ^2^ Department of Neurology S. Giovanni Calibita‐Fatebenefratelli Hospital Rome Italy; ^3^ Clinical Investigation Center INSERM Poitiers University Hospital Poitiers Cedex France; ^4^ Department of Neurology Saint Nazaire Community Hospital Saint‐Nazaire France; ^5^ Department of Vascular Medicine Poitiers University Hospital Poitiers Cedex France

**Keywords:** acute ischemic stroke, patent foramen ovale, right‐to‐left shunt, transcranial Doppler

## Abstract

**Objectives:**

We aimed to confirm the sensitivity and specificity of contrast transcranial Doppler (cTCD) in the detection of right‐to‐left shunt (RLS) compared to the current reference standard (i.e., transesophageal echocardiography—TEE) in patients aged <55 years with a cryptogenic acute ischemic stroke (AIS) or high‐risk (ABCD ^2^ score ≥4) transient ischemic attack (TIA), and to calculate the real life delay in detecting RLS by cTCD versus TEE in a tertiary care academic stroke center.

**Methods:**

Consecutive 16‐ to 54‐year‐old patients with AIS or high‐risk TIA underwent complete diagnostic workup which included, in case of undetermined etiology, cTCD and TEE. Sensitivity and specificity of cTCD, RLS characteristics, and median delay between the two tests were calculated.

**Results:**

Of the 98 included patients, 52 (53%) had a cryptogenic cerebrovascular ischemic event, which displayed a 56% prevalence of RLS related to a patent foramen ovale (PFO) mainly with a high‐grade shunt. When comparing TCD with “bubble test” to TEE, sensitivity and specificity were both 100%. Median delays from symptom onset to examination were 2 (min–max 1–10) and 21 (min–max 1–60) days, respectively, for cTCD and TEE. No adverse event occurred during or after cTDC examination.

**Conclusions:**

Transcranial Doppler with “bubble test” appears as the best screening test for the detection of RLS in young and middle‐aged adults with cryptogenic acute cerebral ischemic events to select patients potentially suitable for closure procedure after TEE confirmation.

## 1 INTRODUCTION

Right‐to‐left shunt (RLS), usually through a patent foramen ovale (PFO), has been implicated in the pathophysiology of acute ischemic stroke (AIS) (Maron, Shekar, & Goldhaber, 2010) and its presence is associated with cryptogenic AIS in patients younger than 55 years (Overell, Bone, & Lees, 2000; Lechat et al., 1988).

Recent studies evaluating recurrence risk in up to 60‐year‐old patients with ischemic stroke of undetermined etiology and PFO found a significant risk reduction after PFO closure associated with long‐term antiplatelet therapy compared to antiplatelet therapy alone (Mas et al., 2017; Søndergaard et al., 2017; Saver et al., 2017).

In the CLOSE trial, a 4.9% five‐year absolute recurrence risk reduction was observed in 16 to 60‐year‐old patients with cryptogenic AIS and PFO associated with atrial septal aneurysm or PFO with large shunt treated by PFO closure plus long‐term antiplatelet therapy compared to antiplatelet therapy alone. In addition, increased risk of new onset atrial fibrillation was found in the closure arm (Mas et al., 2017).

Similarly, in the Gore REDUCE trial, a statistically significant reduction in stroke recurrence was observed in 18‐ to 60‐year‐old patients with cryptogenic AIS and PFO who underwent PFO closure associated with antiplatelet therapy compared with antiplatelet therapy alone. A higher rate of atrial fibrillation and atrial flutter in the interventional arm was observed as well (Søndergaard et al., 2017).

It therefore appears mandatory to ensure a complete workup, which includes the detection of PFO in young and middle‐aged patients with cryptogenic stroke, to accurately select patients who would benefit from the closure procedure.

Transesophageal echocardiography (TEE) is currently considered the reference standard for PFO detection, with a sensitivity and specificity of 100% in an autoptic study (Schneider et al., 1996). However, whether it really has the highest sensitivity in RLS identification has been questioned (Wessler et al., 2015). In addition, TEE is a semi‐invasive examination, not always feasible in a sub‐acute setting.

Contrast transcranial Doppler (cTCD) ultrasound is a noninvasive examination with up to 100% sensitivity for RLS detection compared to TEE, depending on the expertise of the center, the protocol and diagnostic criteria (Sloan et al., 2004).

However, the impact of systematic cTCD assessment as a screening tool for the detection of RLS in young and middle‐aged adults with cryptogenic AIS or high‐risk transient ischemic attack (TIA) needs to be better clarified in a “real‐life” stroke unit setting.

We therefore aimed to assess the prevalence of RLS in our population of patients aged <55 years with a cryptogenic AIS or high‐risk (ABCD^2^ score ≥4) TIA, to confirm the sensitivity and specificity of TCD with “bubble test” in the detection of RLS compared to the current reference standard (i.e., TEE) in patients aged < 5 years with a cryptogenic acute ischemic stroke or high‐risk TIA, and to calculate “real‐life” delay in detecting RLS by cTCD versus TEE after an acute ischemic stroke or high‐risk TIA in a tertiary care academic stroke center.

## 2 METHODS

Consecutive 16‐ to 54‐year‐old patients hospitalized for a radiologically confirmed AIS or high‐risk TIA in our academic tertiary care stroke center from February 2016 to April 2017 were included.

Complete diagnostic workup was assured including brain computed tomography (CT)‐scan with CT‐angiography (CTA) and/or magnetic resonance imaging (MRI) with MR‐angiography (MRA), color‐coded duplex sonography of the neck vessels and transcranial color‐coded duplex sonography, 12‐derivation EKG, at least 48‐hr in‐hospital EKG telemetry, transthoracic echocardiography, extensive laboratory tests including lupus anticoagulant, anticardiolipin antibodies, anti‐beta 2 glycoprotein 1 antibodies, antinuclear antibodies, C‐reactive protein, HIV, Lyme disease and syphilis serologies, and outpatient 24‐hr Holter EKG. All the patients without etiology identified during the acute/early sub‐acute phase underwent in‐hospital cTCD for RLS detection as well as TEE, which was carried out during the hospitalization or in the outpatient period according to cardiologist availability. Shunt degree at the TEE was evaluated based on a 30 microbubble cutoff (mild to moderate ≤30, severe >30) according to an internal protocol. Aortic arch was evaluated by TEE in all patients with cryptogenic stroke or TIA.

Color‐coded duplex sonography of the lower limb veins was performed in all patients with evidence of RLS; however, it often took place after the first 48 hr of hospitalization. Finally, outpatient 14‐day Holter monitoring was planned in case of negative results regarding stroke/TIA etiology, also in patients with identified RLS.

Etiologies, according to the Trial of ORG 10172 in Acute Stroke Treatment (TOAST) (Adams et al., 1993) classification, were reviewed after the extensive workup was completed.

Contrast transcranial Doppler was performed in all patients before TEE, and median delay between the two examinations was calculated. According to standard procedures (Jauss & Zanette, 2000), a TCD 2‐MHz transducer (TCD*X*, Atys Médical, France), fitted on a headband and placed on the temporal bone window, was used to obtain continuous measurement of mean flow velocity in the M1 segment of the middle cerebral artery (MCA). A sample volume of 8 mm in length and a low gain were used to provide a setting optimal for embolus discrimination from the background spectrum. With patients in a supine position, the contrast agent was prepared using 9 ml of isotonic saline solution, 1 ml of air and 1 ml of patient's blood mixed with a three‐way stopcock by exchange of saline/air/blood mixture between the syringes and injected as a bolus in the antecubital vein through an 18‐gauge catheter. High intensity transient signals (HITS), defined as visible and audible short‐duration, high‐intensity signals within the Doppler flow spectrum, were recorded and validated by an experienced neurosonographer (PP). In case of little or no detection of HITS under basal conditions, the examination was repeated using a 10‐s Valsalva maneuver (VM) started 5 s after the injection on the examiner's command. Before the procedure, the patients were trained to perform the VM correctly; the strength of the VM was controlled by peak flow velocity decrease of at least 25% on the Doppler curve. In case of no detection of HITS, the test with VM was repeated twice. The test was considered positive when at least 1 HITS was present within 40 s of the injection (Jauss & Zanette, 2000).

The magnitude of RLS was determined by counting the number of HITS in the MCA in the first 40 s after bolus infusion and by applying both the four‐level visual categorization of the International Consensus Criteria (no occurrence of micro‐embolic signals, grade I: 1–10 signals, grade II: >10 signals but no curtain pattern, grade III: curtain pattern) (Adams et al., 1993) and a simplified version of the Spencer Logarithmic Scale (grade 0: no signals, grade I: 1–10 signals, grade II: 11–30 signals, grade ≥ III: >30 signals) (Spencer et al., 2004).

The study was approved by the local ethics committee, and all enrolled subjects signed informed consent forms.

### 2.1 Statistical analysis

Characteristics between patients with cryptogenic AIS/TIA and patients with acute cerebrovascular ischemic events of determined etiology were compared using the Fisher's exact test and the Wilcoxon two‐sample test for categorical and continuous variables, respectively.

McNemar's test was used to calculate sensitivity and specificity of TCD with “bubble test” compared to contrast TEE, while weighted k coefficient was calculated to compare the International scale and the simplified version of the Spencer scale to the TEE shunt degree.

## 3 RESULTS

**Figure 1:**
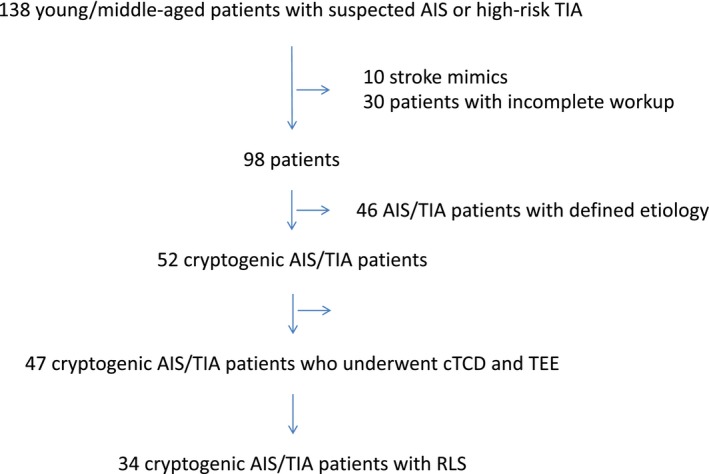
Flow chart presenting the study subjects

A total number of 138 consecutive 16‐ to 54‐year‐old patients hospitalized for a suspected AIS or high‐risk TIA were screened. Forty patients were excluded due to an incomplete diagnostic workup (30 patients) due to death in the acute phase or rapid transfer to the sending secondary care hospital or because of absence of DWI positivity at postacute MRI although long‐lasting symptoms (stroke mimic, 10 patients—Figure 1). Of the 98 included patients (mean age 44 years, *SD* 8, 61% female, 92% Caucasian), in 52 (53%) no clear cerebrovascular ischemic event etiology was found after extensive workup. In this population, TIA represented 15% of subjects in the cryptogenic cerebrovascular ischemic group and 7% in the defined etiology group.

Specific AIS/TIA etiologies according to the TOAST classification in our young and middle‐aged patients are shown in Table 1.

**Table 1:** Acute ischemic stroke/TIA etiologies according to the Trial of ORG 10172 in Acute Stroke Treatment (TOAST) Adams et al., 1993 classification (%)

	16–44 years (*n *= 41)	45–54 years (*n *= 57)
Large‐artery atherosclerosis	2.5	12.3
Cardioembolism	9.8	14
Small‐vessel occlusion	0	8.8
Stroke of other determined etiology	31.7^a^	14^b^
Stroke of undetermined etiology	56	50.9

a Three patients had vertebral artery dissection, two carotid artery dissection, two antiphospholipid syndrome, two essential thrombocytemia, one carotid fibromuscular dysplasia, one carotid aneurysm thrombosis, one neuropsychiatric systemic lupus erythematosus, and one neuro‐Behçet's disease.

b Four patients had carotid artery dissection, one vertebral artery dissection, one carotid aneurysm thrombosis, one catheter angiography complication, one radiation‐induced carotid artery stenosis.

In Table 2, demographic data, vascular risk factors, acute phase treatment information, baseline and discharge clinical severity scores in cerebrovascular ischemic events of undetermined etiology patients compared to TOAST 1 to 4 patients are shown.

**Table 2:** Demographic data, vascular risk factors, acute phase treatment information, baseline and discharge clinical severity scores in patients with cerebrovascular ischemic events of undetermined etiology compared to TOAST 1 to 4 patients

	TOAST 5 (*n *=* *52)	TOAST 1–4 (*n *= 46)	*p* value
Age, years, mean (*SD*)	43 (9)	45 (8)	0.325
Sex, female, *n* (%)	23 (44)	15 (33)	0.300
Arterial hypertension, *n* (%)	8 (15)	15 (33)	0.057
Diabetes, *n* (%)	1 (2)	6 (13)	0.048*
HbA1c, %, mean (*SD*)	5.4 (0.6)	5.7 (1.4)	0.667
Dyslipidemia, *n* (%)	20 (38)	17 (37)	1.000
Total cholesterol, mg/dl, mean (*SD*)	204 (49)	176 (50)	0.012*
LDL cholesterol, mg/dl, mean (*SD*)	126 (45)	109 (42)	0.086
HDL cholesterol, mg/dl, mean (*SD*)	50 (13)	45 (16)	0.030*
Triglycerides, mg/dl, mean (*SD*)	130 (84)	114 (63)	0.345
Smoking habit, *n* (%)	25 (48)	23 (50)	1.000
CAD, *n* (%)	1 (2)	6 (13)	0.048*
Previous CVA, *n* (%)	5 (10)	7 (15)	0.539
PAD, *n* (%)	1 (2)	4 (9)	0.183
OSAS, *n* (%)	2 (4)	7 (15)	0.078
Migraine, *n* (%)	17 (33)	9 (20)	0.368
BMI, mean (*SD*)	27.2 (5.2)	26.3 (7.3)	0.170
Carotid atherosclerosis or increased IMT, *n* (%)	8 (15)	18 (39)	0.001*
IV rt‐PA, *n* (%)	13 (25)	10 (22)	0.812
Thrombectomy, *n* (%)	4 (8)	5 (11)	0.730
Admission NIHSS score, mean (*SD*)	4.3 (6.9)	5.7 (6.6)	0.120
Discharge NIHSS score, mean (*SD*)	1.5 (4.2)	2.9 (6.9)	0.027*
Discharge mRS, mean (*SD*)	0.8 (1.3)	1.5 (1.7)	0.021*
Discharged to home, *n* (%)	47 (90)	34 (74)	0.036*

*Notes*

CAD: coronary artery disease; CVA: cerebrovascular accident; HbA1c: Glycosylated hemoglobin; HDL: high‐density lipoprotein; IMT: intima‐media thickness; IV rt‐PA: intravenous recombinant tissue plasminogen activator; LDL: low‐density lipoprotein; mRS: modified Rankin Score.; NIHSS: National Institutes of Health Stroke Scale; OSAS, BMI: body mass index; PAD: peripheral artery disease; *SD*: standard deviation; TOAST: Trial of ORG 10172 in Acute Stroke Treatment. *p < 0.05.

Forty‐seven cryptogenic AIS/TIA patients underwent both TCD with bubble test and contrast TEE. In five cryptogenic patients, at least one test was not feasible due to inability of the patient to cooperate, while in one patient, cTCD was performed in the basilar artery due to suboptimal temporal bone window, and turned out to be positive with a score of III at the international scale and ≥ III at the Spencer scale. Median delay from symptom onset to examination were 2 (min–max 1–10) and 21 (min–max 1–60) days, respectively, for cTCD and TEE, with a median delay of 17 days (min–max 0–58) between the two examinations.

In 14 TOAST 1‐to‐4 stroke patients, contrast TCD (one patient), contrast TEE (five patients), or both (eight patients) were performed.

In 56% (34/47) of cryptogenic AIS/TIA patients and in 5% (3/14) of patients with defined etiology evaluated for the presence of a RLS, this shunt was detected (*p* 0.001). Right‐to‐left shunt was found to be due to a PFO in all the study subjects except for one, in whom TEE showed an ostium secundum atrial septal defect. Transesophageal echocardiography showed an associated atrial septal aneurysm in 11 of the 34 patients (32%) with a cryptogenic AIS/TIA and RLS and in one of the three patients with defined etiology and RLS. Among the patients with RLS, three (two with cryptogenic stroke and one with a stroke related to an antiphospholipid syndrome) had a distal deep venous thrombosis. However, median delay between symptom onset and color‐coded duplex sonography of the lower limb veins was 4 days.

When comparing TCD with “bubble test” to the currently considered standard reference for cardiac RLS (i.e., TEE) in the global population having undergone both examinations (55 subjects), sensitivity and specificity were both found to be 100%. Among the 34 patients with cryptogenic ischemic acute events and PFO, two had a mild (grade I) shunt according to both the International Consensus Criteria and the simplified version of the Spencer Scale, six and one patients had a grade II shunt according to the International Consensus Criteria and the Spencer Scale, respectively, and 26 patients had a grade III shunt according to the International scale while 31 patients had a grade ≥ III on the simplified version of the Spencer Scale. Comparing both cTCD scales to the TEE shunt grading, weighted K coefficients were 0.96 and 0.99 for the international scale and for the simplified Spencer scale, respectively.

Finally, no adverse event occurred during or after cTDC examination.

## 4 DISCUSSION

In this study, a high proportion of unknown etiology was observed in young and middle‐aged adult patients with acute ischemic stroke or high‐risk TIA after extensive workup. This high percentage was comparable to previously published research demonstrating that half of strokes in young patients are cryptogenic (Amarenco, 2005).

In our population, patients with cerebrovascular acute ischemic events of undetermined etiology were less frequently diabetic and had a higher HDL cholesterol level and a trend toward lower arterial hypertension prevalence compared to patients with a defined etiology. Interestingly, total cholesterol level was higher in this subgroup of patients, while coronary artery disease was less frequent. Cryptogenic stroke showed no or minimal atherosclerotic changes as localized intima‐media thickening or small atherosclerotic plaques. A trend toward lower OSAS prevalence was observed, however, without statistical significance.

Similar data were obtained in a population‐based study in Oxfordshire, showing that patients with cryptogenic AIS or TIA had the lowest prevalence of risk factors for atherosclerosis, the lowest frequency of comorbid atherosclerotic disease, and the lowest risk of acute coronary events compared with patients with events of known cause (Li et al., 2015).

In our study population, clinical severity at hospital admission did not statistically differ between the two groups while in‐hospital clinical evolution appeared more favorable in the cryptogenic stroke/TIA group as demonstrated by lower National Institutes of Health Stroke Scale (NIHSS) score and modified Rankin Score (mRS) at discharge as well as a higher proportion of patients discharged home in this group compared to the defined etiology group.

In the subgroup of patients with cryptogenic cerebral ischemic events, 56% RLS prevalence was detected. Although the small proportion of patients with stroke/TIA of determined etiology having undergone RLS shunt evaluation does not enable accurate comparison of shunt prevalence in our population, a significantly higher proportion of RLS was found in our cryptogenic stroke/TIA patients compared to TOAST 1 to 4 patients. This finding is consistent with previously published studies, which demonstrated prevalence of 44% to 66% of PFO in patients with cryptogenic stroke as compared to 10%–27% in general population and 21%–33% in patients with stroke of defined etiology (Lechat et al., 1988; Hagen, Scholz, & Edwards, 1984; Steiner et al., 1998; Job et al., 1994). In a series of 95 patients with first ischemic stroke over age 39 evaluated by TEE, Steiner and colleagues found 45% frequency of PFO among patients with cryptogenic infarcts compared to patients with determined cause of stroke (23%, *p* = 0.02) (Steiner et al., 1998). In a population of 60 adults under 55 years old with ischemic stroke and a normal cardiac examination, significantly higher prevalence of PFO was found in patients with stroke (40%) than in the control group (10%, *p* < 0.001), with higher prevalence (54%) in patients with no identifiable cause compared to those with a defined etiology (21%) (Lechat et al., 1988). Similar differences in proportion of PFO prevalence were found in a series of 137 subjects with 66% prevalence in patients with unclarified compared to 33% in clarified stroke etiology, while a very high proportion (43%) of PFO, mainly of low degree, was found in normal subjects (Job et al., 1994).

Furthermore, we confirm that cTCD is an extremely accurate examination for detection of RLS with sensitivity and specificity of 100% in our series compared to the currently considered standard reference. In a systematic review of the literature of 27 prospective studies assessing intracardiac RLS using contrast TCD compared with contrast TEE on a total number of 1,968 patients, sensitivity and specificity of TCD with bubble test were 97% and 93%, respectively (Mojadidi et al., 2014). However, accuracy may vary by center, protocol, and diagnostic criteria, with sensitivity ranging from 70% to 100% and specificity >95%, as estimated by the subcommittee of the American Academy of Neurology (Wessler et al., 2015). Furthermore, one can raise the question as to whether TEE is the real gold standard for RLS detection as in some cases, it failed to detect an existing shunt (Rodrigues et al., 2013; Caputi et al., 2009; Maillet et al., 2018), and a few studies have shown even higher sensitivity of cTCD compared to TEE (Wessler et al., 2015; Maillet et al., 2018; Tobe, Bogiatzi, Munoz, Tamayo, & Spence, 2016), which is probably at least partially due to difficulty in correctly performing the Valsalva maneuver during TEE. In our opinion, TCD with “bubble test” performed according to rigorously standardized procedure should be ensured to all young and middle‐aged cryptogenic acute ischemic stroke patients as the screening test for RLS detection given the fact that it is extremely accurate, noninvasive, low expensive, rapidly available and safe. In case of a positive result, TEE must be performed to confirm the localization of the shunt at the cardiac level (taking into account the infrequent cases of pulmonary arteriovenous fistulas), to evaluate whether the shunt is due to a PFO or to another atrial septal defect, and to detect frequently associated atrial septal aneurysm. Furthermore, frequent delay in obtaining TEE confirms the usefulness of cTCD in current clinical practice, as a way of selecting patients who would derive maximum benefit from TEE and of detecting those who would potentially be suitable for closure procedure.

Regarding shunt degree, in the majority of our cryptogenic AIS/TIA patients with PFO, a large shunt was found, with high concordance between cTCD and cTTE shunt grading. Both the International scale (Jauss & Zanette, 2000) and the simplified version of the Spencer scale (Spencer et al., 2004) appeared to accurately predict shunt degree, with possibly higher cutoff limit specificity at 30 microembolic signals for large shunts, as previously suggested by Lao and colleagues (Lao et al., 2008).

Our study has several limitations. First, included patient age limit was <55 years while recent interventional studies included patients up to 60 years old. In the present study, this limit was chosen to allow evaluate young and middle‐aged adults with acute cerebral ischemic events, however, extension to 60‐year‐old patients would be appropriate. Second, color‐coded duplex sonography of the lower limb veins was performed during hospitalization often after the first 48 hr. We therefore cannot discriminate whether those few cases of DVT were responsible for a paradoxical embolism or were a stroke complication. In fact, the paradoxical embolism is considered the cause of RLS‐related stroke, although this has rarely been demonstrated. Third, transthoracic echocardiography was often performed without injection of agitated saline contrast; therefore, it was not possible to assess its accuracy in detecting RLS in the present study. However, lower sensitivity compared to the cTCD has already been clearly demonstrated. Forth, formal sleep study was not performed in all the study subjects; presence of OSAS was otherwise established on reported previous diagnosis or on clinical suspicion. The trend toward higher prevalence of OSAS in patients with cryptogenic compared to TOAST 1‐to‐4 AIS/TIA patients, which remains without overt significance, should be more accurately verified.

In conclusion, contrast transcranial Doppler appears as the best screening test for the detection of RLS in young and middle‐aged adults with acute cerebral ischemic events of undetermined etiology to select those who could be suitable for closure procedure after TEE confirmation.

## CONFLICT OF INTEREST

None.
